# Recirculating glass pipettes constitute a high risk when working with freshly isolated immune cells - the presence of bacterial pyrogenic material

**DOI:** 10.3389/fcimb.2025.1689489

**Published:** 2025-11-21

**Authors:** Srinivas Akula, Sandra Lara, Anna-Karin Olsson, Lars Hellman

**Affiliations:** 1 Department of Cell and Molecular Biology, Uppsala University, The Biomedical Center, Uppsala, Sweden; 2 Department of Medical Biochemistry and Microbiology, The Biomedical Center (BMC), Uppsala, Sweden

**Keywords:** monocytes, macrophages, cell lines, lipopolysaccharides, glass pipettes, inflammation

## Abstract

A number of immune cells are highly sensitive to pyrogenic substances such as bacterial lipopolysaccharides (LPS). It is therefore crucial to ensure that all material coming in contact with these cells are pyrogen free. We here present a comparative analysis of the induction of inflammatory cytokines by freshly isolated human peripheral blood monocytes when handling the cells with recirculating glass pipettes or disposable plastic pipettes. The glass pipettes were sterilized but previously used for multiple projects including work with bacteria. Using these glass pipettes resulted in the same induction of a set of inflammatory cytokines and chemokines as when adding 1 ug of LPS/ml to the culture medium, an exceptionally high level of LPS. The most extreme upregulation was seen for IL-6, which increased in expression by a factor of more than 75 000-fold already by four hours of *in vitro* culture indicating that great care should be taken when using glass pipettes that are recycled in the lab for culturing of eukaryotic cells. IL-8 became the most highly expressed gene by four hours incubation exceeding the previous top transcript, which was lysozyme, by 50%. This finding clearly shows that glass pipettes, despite the careful washing procedures and sterilizing during recirculation, is not advisable to use when working with cells, in particular freshly isolated immune cells. Based on these data it seems as pyrogen free plastic pipettes, in spite of the fact that this involves an increase in plastic disposal, is the only acceptable solution to obtain biologically relevant results. A possible alternative could be to use glass pipettes that are used only for work with eukaryotic cells and that these pipettes are kept in containers with anti-microbial substances to avoid any microbial growth during storage in the lab between washings. However, such procedures need to be carefully tested and monitored during long term use.

## 1 Introduction

To use freshly isolated eukaryotic cells, and also cell lines, during *in vitro* culture conditions involves a number of handling steps, such as culture flasks, culture media, serum as well as other additives. The risk is always that some of these components act as strong activators of the cells. A strong adherence to a culture flask may for example result in phagocytic behavior of many inflammatory cells, such as monocytes, macrophages and neutrophils, and the induction of an inflammatory response. Pyrogenic substances, such as lipopolysaccharides, peptidoglycan fragments, fimbriae fragments or bacterial products, in culture media, serum or possibly also on recirculating glass pipettes may also result in a massive inflammatory response in these cells, which may affect the results of the experiments. It is therefore essential to ensure that the culture conditions as much as possible mimic the *in vivo* condition. However, this is not always an easy task. For both economical and environmental reasons, there is a wish to avoid disposable plastic ware. As a consequence, there is a substantial risk of contamination with highly activating substances such as pyrogenic substances originating from bacteria. The type of culture flasks used during *in vitro* culture can also be of major importance to limit adherence and thereby an inflammatory response. In addition to the actual culture conditions as a confounding factor when interpreting data from cultured cells comes also the use of relatively non-physiological model systems, such as several of the commonly used cell lines. Many of them show little resemblance to their *in vivo* counterparts, and should therefore as much as possible be avoided as models for the biological function of cells *in vivo*.

During a recent project where we analyzed the response to *E. coli* LPS by human peripheral blood monocytes we observed almost no difference in response when adding 1 ug/ml of LPS to the culture (Lara et al., 2022). We then changed to plastic pipettes, which resulted in no or only minimal inflammatory cytokine and chemokine response by these monocytes, clearly indicating that it was the glass pipettes that was the major problem. To look closer into this issue we have here extended this analysis by performing several experiments during an extended period of time involving several studies over a 3-year time period. We have analyzed freshly isolated human peripheral blood monocytes during *in vitro* culture for four hours. In one part of the experiment we used recirculating glass pipettes and in the second part pyrogen free plastic pipettes and performed a genome-wide analysis of inflammatory gene expression. In these experiments we also used a new type of culture flasks that minimize the adherence of freshly isolated human monocytes. The data we obtained was more extreme than we had expected and in line with the first experiments in the previous study of the response by human blood monocytes to LPS (Lara et al., 2022). The use of washed and sterilized glass pipettes that are recycled in the lab, and therefore used in both eukaryotic and prokaryotic lab work, resulted in a massive increase in the expression levels of a distinct set of inflammatory cytokines and chemokines. We feel that this information is essential for all scientists working with cell biology by studies of cells in *in vitro* culture and in particular for all immunologists working with inflammatory cells, such as monocytes, macrophages and neutrophils but also other immune cells that respond strongly to pyrogenic substances.

## 2 Materials and methods

### 2.1 Washing procedure of glass pipettes

The glass pipettes were washed in a machine designed for pipettes and with a special detergent. The machine, named PSD, was from GEWO Feinmechanik Gmbh, Hörlkofen, Germany and the detergent (Labwash Premium extra PF) from VWR International Leuven, Belgium. The washing included several steps with water and with detergent at 90°C according to the standard procedure recommended by the manufacturer. The pipettes were then heat sterilized for 4 hours at 180°C.

### 2.2 Purification of monocytes from human peripheral blood

Peripheral blood monocytes were isolated from buffy coats, from healthy anonymous donors at the Akademiska Hospital in Uppsala, Sweden. Mononuclear cells (PBMCs) were isolated by standard density gradient centrifugation using Ficoll–Paque Plus (GE Healthcare, Uppsala, Sweden). PBMCs were further washed with PBS containing 2 mM of EDTA, and incubated with anti-CD14 coated magnetic beads (Miltenyi Biotec, Bergisch Gladbach, Germany). Positive selection of CD14^+^ cells was performed through magnetic cell separation. Subsequently, CD14 cells were stained with anti-human CD14 PE antibody (clone: 61D3, Invitrogen, Carlsbad, CA, USA) and the purity was verified (over 96%) by flow cytometry.

Four million of these cells were immediately flash frozen in liquid nitrogen and stored at -80°C for preparation of total RNA. The remaining cells were transferred into six different culture flasks with approximately 2.5 million cells per flask. We used the Cellstar culture flasks with a cell-repellent surface, developed for minimal activating properties, white filter screw cap sterile 50 ml (25cm^2^) (Greiner Bio-One GmbH, Kremsmünster, Austria, product number 690985). One culture flask was used to culture cells for 4 hours in RPMI-1640 with 10% fetal bovine serum (FBS), without addition of any immuno-stimulant. One flask was used to culture cells for four hours with 1 ug/ml of *Escherichia coli* LPS (Sigma -Aldrich L4516- from *E. coli* O127:B8). Two identical sets of cells were analyzed, one where we used recirculating glass pipettes and one where we used disposable pyrogen free plastic pipettes.

### 2.3 Analysis of the total transcriptome

Total RNA was prepared from the CD14^+^ monocytes, both the freshly isolated and the cultured cells from each donor, using the RNeasy Plus mini kit (Qiagen, Hilden, Germany), according to the manufacturer´s recommendations. The RNA was eluted with 30 μl of DEPC-treated water, and the concentration of RNA was determined by using a Nanodrop ND-1000 (Nano Drop Technologies, Wilmington, Delaware, USA). Later the integrity of the RNA was confirmed by visualization on 1.2% agarose gel using ethidium bromide staining.

The transcriptome of freshly isolated and cultured cells of the four different donors were analyzed for their total transcriptome by the Thermo Fisher chip-based Ampliseq transcriptomic platform at the SciLife lab in Uppsala, Sweden. The sequence results were delivered in the form of Excel files with normalized expression levels for an easy comparison of expression levels between samples.

Three years later this experiment was repeated with two donors. However, this time the transcriptome was analyzed by RNA-seq methodology at the SciLife lab in Uppsala and the reads were transferred into a Excel-file for direct comparison with previous Ampliseq data.

## 3 Results

### 3.1 Purification of monocytes from human peripheral blood

Monocytes were purified from buffy coats, from six different donors. The last two samples were purified three years later than the initial four, so the results have been replicated over a period of three years with essentially the same results. The cells were first washed in PBS and then subjected to a two-step purification using density gradient centrifugation followed by magnetic bead separation using a monoclonal anti-human CD14 antibody. 96-98% pure population of blood monocytes from all six donors were obtained following this two-step purification protocol. Approximately four million of these cells from each of the six donors were immediately pelleted and total RNA was purified by a standard protocol.

### 3.2 *In vitro* culture of purified peripheral blood monocytes

**Figure 1:**
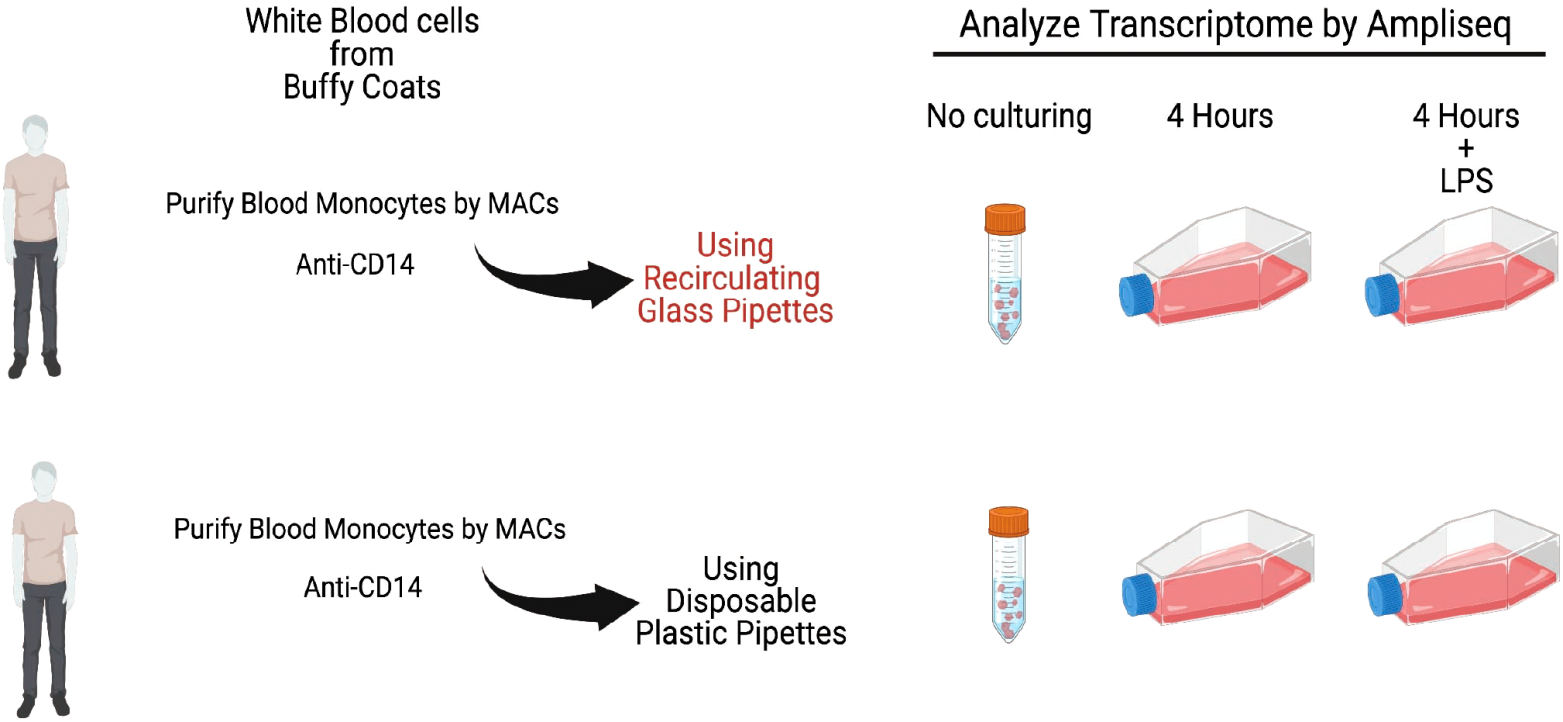
A schematic representation of the outline of the experiment. Monocytes from six individual persons were analyzed. One set of cells were frozen directly after purification as time point 0. The remaining cells were divided into equal fractions and put into separate flasks for *in vitro* culturing for 4 hours with or without LPS. Following the culturing total RNA was purified from all cell samples including the 0 time point for further analysis by Ampliseq or RNA-seq to study their entire transcriptome.

The remaining cells from each of the donors were divided into culture dishes, with an equal number of cells, approximately 4 million cells per culture dish. One set of the cultures from each of the donors were grown in the presence of only cell culture medium during four hours. For all six of the donors an identical dish was cultured for four hours with the addition of 1 ug of *E. coli* LPS/ml to the culture medium. For the cultures from four of the donors we used recirculated glass pipettes when adding culture medium and cells, and for the two other donors we used disposable pyrogen free plastic pipettes (see **Figure 1** for an experimental overview). The glass pipettes had been washed at a central facility in a machine designed for pipette washing and with a specifically designed detergent mix. Following the careful washing procedure involving multiple steps with water and with detergent at 90°C the pipettes were heat sterilized for 4 hours at 180*°*C.

Following the *in vitro* culturing, the cells were harvested and total RNA was prepared from each of the different cultures and subjected to analysis of their transcriptome.

### 3.3 Ampliseq and RNA-seq analysis of the total transcriptome of the different monocyte samples

Total RNA from the untreated cells and from the first four set of cultures were analyzed by the Thermo Fisher Ampliseq technology. The result was delivered in the form of Excel files with normalized reads for the separate transcripts. The samples from the last two individuals were analyzed by RNA-seq technology. We have thereby used two different technologies to analyze the transcriptome and also separated the two set by over a three-year period as the first four set of cultures were performed in 2022 and the last two cultures in spring 2025. We selected the four inflammatory cytokines, IL-1α, IL-1β, IL-6 and TNF-α and the two inflammatory chemokines IL-8 and CCL4, since these were the cytokines and chemokines that showed the most pronounced upregulation by LPS from a previous study (Lara et al., 2022).

**Figure 2:**
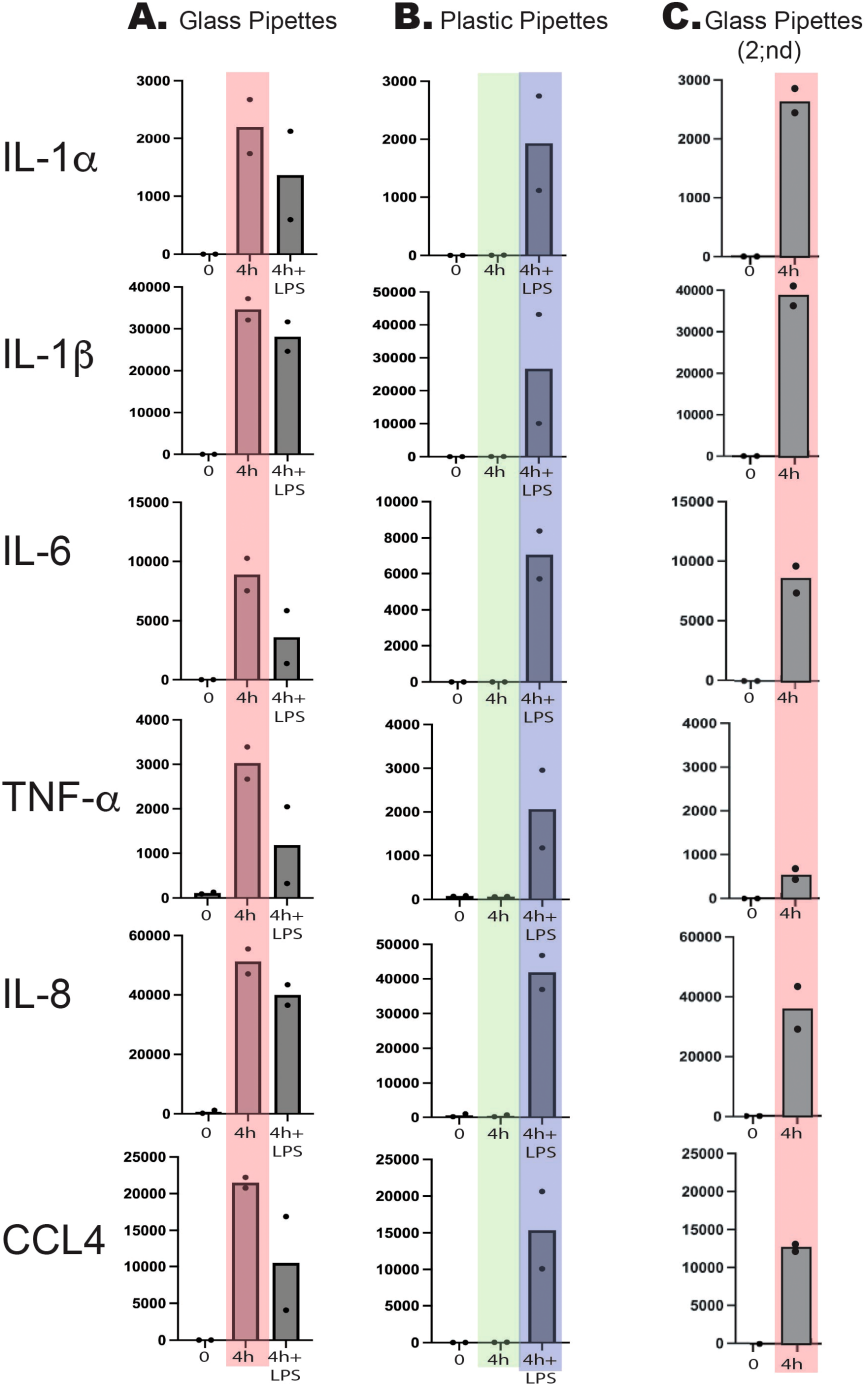
Total reads of six different markedly upregulated genes at two timepoints, directly after purification and after four hours of *in vitro* culture in the presence or absence of bacterial LPS (1 ug/ml). Two sets of experiments were performed with two individuals in each experiment. In the first experiment we used recirculating glass pipettes **(A)** and in the second disposable pyrogen free plastic pipettes **(B)**. The major difference between the two experiments is seen at four hours in the absence of added bacterial LPS. These two sets of values are marked light red for the glass pipettes and in light green for the plastic pipettes. As can be seen from the figure glass pipettes contain sufficient amounts of LPS or other pyrogenic substances from bacteria to give similar activation or even higher than after addition of 1 ug/ml of LPS (marked in light blue). Three years later this experiment was repeated with two sets of cells directly isolated and cultivated for four hours after being handled with glass pipettes (Glass Pipettes 2;nd, **(C)** with essentially the same result showing the high content of pyrogenic substances in recirculating glass pipettes over a three-year period.

As can be seen from **Figure 2**, a strong upregulation of all of these six cytokines and chemokines was induced simply from using recirculating glass pipettes. This upregulation was in the same range as after addition of 1 ug LPS to the culture medium. No additional increase was seen after adding 1 ug/ml to these cultures, indicating that the maximal level of cell activation had already been reached by using the recirculating glass pipettes (**Figure 2**). In one of the samples, we detected a marked decrease in the IL-1α, IL-6, TNF-α and CCL4 responses, indicating an overstimulation by excessive amounts of LPS (**Figure 2**). This shows that even after careful washings and sterilizing of the pipettes, they still contain LPS or other bacterial pyrogenic substances to a level sufficient to deliver an amount of LPS in the range of 1 ug/ml or higher by a single transfer of culture media. In addition to LPS the pipettes probably also contained peptidoglycan fragments and possibly also fimbriae fragment, which similar to LPS also can trigger signaling from toll like receptors (TLRs) and/or NOD1/NOD2.

We analyzed the LPS content of the cell culture medium after using glass pipettes by a commercial LPS-kit (Pierce chromogenic Endotoxin quant kit from Thermo Fisher). To our surprise we found relatively low levels of LPS indicating that the LPS either is modified after autoclaving to such an extent that the kit fails to detect it or that the majority of pyrogenic substances are bacterial substances other than LPS. To confirm the results from the initial analysis we therefore performed an analysis of a new set of monocytes for 4 hours using glass pipettes and found the same massive upregulation of the same set of classical inflammatory as shown in **Figure 2**. This analysis which was performed three years later than the first set as described above confirms the results from the first set of cultures but still leaving us with the lack of identity of the pyrogenic substances. However, the almost exact response concerning level and type of cytokines between glass pipettes and LPS indicates that the response in both situations act through TLR-4, which is the main receptor for *E. coli* LPS.

## 4 Discussion

Monocytes, macrophages and also neutrophils are extremely sensitive to even small amounts of pyrogenic substances, such as bacterial LPS. To obtain reliable results when studying these cells, it is crucial to minimize the risk of contaminating the cultures with pyrogenic substances. We have recently shown that human monocytes react very rapidly and very strongly to LPS by upregulating a limited set of inflammatory cytokines and chemokines (Lara et al., 2022). The cytokines with the most pronounced upregulation were essentially only the traditional inflammatory cytokines IL-1α, IL-1β, IL-6 and TNF-α. Among the chemokines it was IL-8 and CCL4 that were the most highly upregulated transcripts. In these activated monocytes, IL-8 actually becomes the dominating transcript after only four hours in the presence of LPS and thereby without comparison most highly expressed transcript of the activated monocyte (Lara et al., 2022). We therefore selected this set of six cytokines and chemokines to study the effect of using recirculating glass pipettes during handling and culturing of freshly isolated human blood monocytes. To avoid interference by plastic adherence we used a new type of culture flasks optimized for low activation of cells. The plastic coating of these culture flasks results in that the absolute majority of the cells stayed non-adherent even after 24 and 48 hours in culture, much better mimicking the *in vivo* conditions compared to previous culture flasks. By using these type of culture flasks, we did not observe any major increase in any of the inflammatory cytokines and chemokines during culturing in the absence of LPS and using pyrogen free plastic pipettes (**Figure 2**). However, when using recirculating glass pipettes the upregulation of these six inflammatory cytokines and chemokines were in the same range as when adding 1 ug of E. coli for LPS per ml to the culture medium (**Figure 2**). After four hours in culture IL-1α was upregulated 15000 times, IL-1β 3380 times, IL-6–75000 times, TNF-α 37 times, IL-8–320 times and CCL4–6900 times (**Figure 2**) (Lara et al., 2022). These data clearly show the danger of using recirculating glass pipettes during culturing of mammalian cells and in particular cells that are the infection sensing cells of the immune system, such as monocytes, macrophages and neutrophils. However, most cell lines are likely also markedly affected by the presence of pyrogenic substances.

LPS and fimbriae from *E. coli* primarily stimulates monocytes through TLR-4, whereas peptidoglycan fragments act most likely through the intracellular sensors NOD1/NOD2 (Frendeus et al., 2001; Travassos et al., 2004). The Monocytes were purified based on the presence of CD14, which acts together with MD-2 as coreceptors for LPS on the monocytes. The signaling from TLR-4 goes through the adaptor molecules Mal and MyD88 (Fitzgerald et al., 2001) LPS binds to the LPS binding protein (LBP) which is found in the circulation. This complex binds to CD14, which together with MD-2 transfer the LPS to TLR-4 (Maeshima and Fernandez, 2013). We checked the expression levels of CD14, TLR-4 and MyD88 before and after LPS stimulation of the monocytes. CD14 expression is reduced from around 1700 counts to 600 counts after 4 hours and then increases 3–5 fold after 24–48 hours of stimulation to approximately 6000 counts by 24 hours and to 8000 by 48 hours. TLR-4 increases dramatically by 4 hours from 24 counts to over 600, but then decreases by 24 and 48 hours to around 130 counts and thereby a 5-fold increase from base line levels. In contrast MyD88 levels only change quite modestly from 90 to 200 counts by 4 hours to return to around 90 counts by 24 to 48 hours in the presence of LPS. Thus, we see changes also in the transcription of the genes involved in the response to LPS, and that these changes are quite dynamic during the first 48 hours of LPS stimulation.

The lack of detection of LPS in significant amounts with the commercial LPS kit indicates that great care should be taken to rely exclusively on such kits as the freshly isolated monocytes apparently reacted extremely strongly. This indicates that pyrogenic material still may be present even if the commercial test indicates the absence. The reason for this is still not known but may originate in modification of the bacterial pyrogenic substances by the high temperatures used during the autoclaving step during pipette cleaning. The most accurate test is thereby probably to use freshly isolated monocytes as we here have done and that is used in the validated Monocyte activation test (MAT) that is present in the European Pharmacopedia. The MAT has to a large extent replaced the rabbit pyrogen test. The major difference between our test and the MAT is that we have used transcriptomal analysis to look at all potential upregulated genes, whereas the MAT often use IL-1b and IL-6 analyzed by ELISA as read outs (da Silva et al., 2016; Gimenes et al., 2024). These two cytokines were actually the ones we could detect the highest increase of in the transcriptome. We shall here also add that there are a number of other kits on the market which could be used as alternative to test for pyrogenic material, such as the Limulus test and that they show differences in sensitivity to different pyrogenic substances as discussed in the article by da Silva et al (da Silva et al., 2016). and Spoladore et al (Spoladore et al., 2021).

When studying mammalian cells *in vitro* there are a number of difficulties that need to be overcome. The culture media and the serum need to mimic *in vivo* conditions as much as possible with respect to pH, salt concentration and nutrient composition. The atmosphere in the incubator should also give CO_2_ and oxygen concentrations that reflect the *in vivo* conditions. To this comes the importance of avoiding contaminating the cultures with pyrogenic substances during handling and culturing the cells. We can here show that using glass pipettes that are not exclusively used for handling mammalian cells involves a major risk for obtaining erroneous data. It is not unlikely that a significant proportion of published studies reporting various responses by purified cells and cell lines are more or less affected by pyrogenic substances from pipettes and/or contaminated media or sera. Another source of potential error is the use of cells and cell lines that do not correspond to the type of cell they are meant to represent. We have recently analyzed both *in vitro* differentiated mouse bone marrow-derived mast cells and human monocytic cell lines for their transcriptomes and found that they differ to very large extent from their *in vivo* counterparts (Akula et al., 2020; Akula et al., 2022). In addition, when we analyzed the transcriptome of the cell line THP-1, commonly used as a model for human monocyte biology, we found very little resemblance to freshly isolated human blood monocytes (Akula et al., 2022). Instead, the transcriptome of THP-1 was more similar to early neutrophilic granulocytes (Akula et al., 2022). When screening PubMed for articles using THP-1 as model system we found 14–500 hits. If a large fraction of these publications involved using THP-1 as a monocyte model the results may be totally misleading. The same situation is now also valid for the use of glass pipettes. A very large number of studies may have given completely erroneous results as the cells may already have been fully activated before the test substances aimed to be analyzed was added, as we observed during the initial studies of LPS activation of human monocytes. We do not accuse the authors of these studies to deliberately have manipulated data. However, the use of poorly characterized cells and cell lines and culture conditions such as adhesive plastic ware or glass pipettes that contain high levels of pyrogenic material may have resulted in the publication of data that by no means reflect the *in vivo* situation or in worst case be totally erroneous.

In summary, the high amounts of pyrogenic substances in recirculating glass pipettes, shows that great care should be taken when studying mammalian cells during *in vitro* culture conditions. *In vivo* studies are almost always the best alternative when analyzing various cell biological processes. However, many studies cannot be performed *in vivo* and such studies are extremely costly and time consuming, why *in vitro* cultures are often the only possible alternative. However, great care should be taken when performing such studies with respect to culture conditions and the selection of cell system to avoid obtaining results that have little relevance for the *in vivo* situation. The information presented here concerning the danger of using recirculating glass pipettes in various studies of cell biology is therefore essential for all scientist working with cell biology and particularly with inflammatory immune cells, if the aim is to obtain biologically relevant information.

## Data Availability

The original contributions presented in the study are included in the article/supplementary material, further inquiries can be directed to the corresponding author/s.

## References

[B1] Akula S. Lara S. Olsson A. K. Hellman L. (2022). Quantitative analysis of the transcriptome of two commonly used human monocytic cell lines-THP-1 and mono mac 6-reveals their arrest during early monocyte/neutrophil differentiation. Int. J. Mol. Sci. 23. doi: 10.3390/ijms23105818, PMID: 35628628 PMC9145822

[B2] Akula S. Paivandy A. Fu Z. Thorpe M. Pejler G. Hellman L. (2020). How relevant are bone marrow-derived mast cells (BMMCs) as models for tissue mast cells? A comparative transcriptome analysis of BMMCs and peritoneal mast cells. Cells 9. doi: 10.3390/cells9092118, PMID: 32957735 PMC7564378

[B3] da Silva C. C. Presgrave O. A. Hartung T. de Moraes A. M. Delgado I. F. (2016). Applicability of the Monocyte Activation Test (MAT) for hyperimmune sera in the routine of the quality control laboratory: Comparison with the Rabbit Pyrogen Test (RPT). Toxicol. In Vitro. 32, 70–75. doi: 10.1016/j.tiv.2015.12.004, PMID: 26688320

[B4] Fitzgerald K. A. Palsson-McDermott E. M. Bowie A. G. Jefferies C. A. Mansell A. S. Brady G. . (2001). Mal (MyD88-adapter-like) is required for Toll-like receptor-4 signal transduction. Nature. 413, 78–83. doi: 10.1038/35092578, PMID: 11544529

[B5] Frendeus B. Wachtler C. Hedlund M. Fischer H. Samuelsson P. Svensson M. . (2001). Escherichia coli P fimbriae utilize the Toll-like receptor 4 pathway for cell activation. Mol. Microbiol. 40, 37–51. doi: 10.1046/j.1365-2958.2001.02361.x, PMID: 11298274

[B6] Gimenes I. Spoladore J. Paranhos B. A. Romasco T. Di Pietro N. Piattelli A. . (2024). Assessment of pyrogenic response of medical devices and biomaterials by the monocyte activation test (MAT): A systematic review. Int. J. Mol. Sci. 25. doi: 10.3390/ijms25147844, PMID: 39063086 PMC11276646

[B7] Lara S. Akula S. Fu Z. Olsson A. K. Kleinau S. Hellman L. (2022). The human monocyte-A circulating sensor of infection and a potent and rapid inducer of inflammation. Int. J. Mol. Sci. 23. doi: 10.3390/ijms23073890, PMID: 35409250 PMC8999117

[B8] Maeshima N. Fernandez R. C. (2013). Recognition of lipid A variants by the TLR4-MD-2 receptor complex. Front. Cell Infect. Microbiol. 3, 3. doi: 10.3389/fcimb.2013.00003, PMID: 23408095 PMC3569842

[B9] Spoladore J. Gimenes I. Bachinski R. Negherbon J. P. Hartung T. Granjeiro J. M. . (2021). Standardized pyrogen testing of medical products with the bacterial endotoxin test (BET) as a substitute for rabbit Pyrogen testing (RPT): A scoping review. Toxicol. In Vitro. 74, 105160. doi: 10.1016/j.tiv.2021.105160, PMID: 33831473

[B10] Travassos L. H. Girardin S. E. Philpott D. J. Blanot D. Nahori M. A. Werts C. . (2004). Toll-like receptor 2-dependent bacterial sensing does not occur via peptidoglycan recognition. EMBO Rep. 5, 1000–1006. doi: 10.1038/sj.embor.7400248, PMID: 15359270 PMC1299148

